# A New Risk Prediction Model for Venous Thromboembolism and Death in Ambulatory Lung Cancer Patients

**DOI:** 10.3390/cancers15184588

**Published:** 2023-09-15

**Authors:** Patricia Gomez-Rosas, Cinzia Giaccherini, Laura Russo, Cristina Verzeroli, Sara Gamba, Carmen Julia Tartari, Silvia Bolognini, Chiara Ticozzi, Francesca Schieppati, Luca Barcella, Roberta Sarmiento, Giovanna Masci, Carlo Tondini, Fausto Petrelli, Francesco Giuliani, Andrea D’Alessio, Mauro Minelli, Filippo De Braud, Armando Santoro, Roberto Labianca, Giampietro Gasparini, Marina Marchetti, Anna Falanga, on behalf of the HYPERCAN Investigators

**Affiliations:** 1 Immunohematology and Transfusion Medicine, Hospital Papa Giovanni XXIII, 24127 Bergamo, Italy; patmlhs@hotmail.com (P.G.-R.); cverzeroli@asst-pg23.it (C.V.); sbolognini@asst-pg23.it (S.B.); chiaraticozzi97@gmail.com (C.T.); fschieppati@asst-pg23.it (F.S.); annafalanga@yahoo.com (A.F.); 2 Cardiovascular Research Institute Maastricht (CARIM), Maastricht University Medical Center (MUMC+), 6229 ER Maastricht, The Netherlands; 3 Hospital de Oncologia, Unidad Medica de Alta Especialidad (UMAE), Centro Medico Nacional Siglo XXI, Instituto Mexicano del Seguro Social (IMSS), Mexico City 06720, Mexico; 4 Oncology Unit, Hospital San Filippo Neri, 00135 Rome, Italy; 5 Oncology Unit, IRCCS Humanitas Institute, 20089 Rozzano, Italy; 6 Oncology Unit, ASST Papa Giovanni XXIII, 24127 Bergamo, Italy; 7 Oncology Unit, Hospital Treviglio-Caravaggio, 24047 Treviglio, Italy; 8 Oncology Unit, IRCCS Cancer Institute Giovanni Paolo II, 70124 Bari, Italy; 9 Medical Oncology and Internal Medicine, Policlinico San Marco, Gruppo San Donato Zingonia-Bergamo, 24046 Zingonia, Italy; 10 Oncology Unit, Hospital San Giovanni Addolorata, 00184 Rome, Italy; 11 Oncology Unit, IRCCS National Cancer Institute, 20133 Milan, Italy; 12 Fondazione ARTET Onlus, 24121 Bergamo, Italy; rlabian@tin.it; 13 School of Medicine and Surgery, University of Milan Bicocca, 20126 Milan, Italy

**Keywords:** non-small cell lung cancer, venous thromboembolism, hypercoagulability, biomarkers, survival, D-dimer, thrombin generation, risk assessment model

## Abstract

**Simple Summary:**

The predictive value of existing venous thromboembolism risk assessment models (RAMs) in lung cancer patients is still debated, and the design of new models represents an unmet clinical need. In a prospective cohort of patients with newly diagnosed metastatic lung cancer, clinical characteristics, and hemostatic biomarkers assessed before initiating chemotherapy were used to generate a more accurate RAM. This easy-to-implement RAM was compared to four previously published scores, which were also externally validated in this study.

**Abstract:**

(1) Background: Venous thromboembolism (VTE) is a frequent complication in ambulatory lung cancer patients during chemotherapy and is associated with increased mortality. (2) Methods: We analyzed 568 newly diagnosed metastatic lung cancer patients prospectively enrolled in the HYPERCAN study. Blood samples collected before chemotherapy were tested for thrombin generation (TG) and a panel of hemostatic biomarkers. The Khorana risk score (KRS), new-Vienna CATS, PROTECHT, and CONKO risk assessment models (RAMs) were applied. (3) Results: Within 6 months, the cumulative incidences of VTE and mortality were 12% and 29%, respectively. Patients with VTE showed significantly increased levels of D-dimer, FVIII, prothrombin fragment 1 + 2, and TG. D-dimer and ECOG performance status were identified as independent risk factors for VTE and mortality by multivariable analysis and utilized to generate a risk score that provided a cumulative incidence of VTE of 6% vs. 25%, death of 19% vs. 55%, and in the low- vs. high-risk group, respectively (*p* < 0.001). While all published RAMs significantly stratified patients for risk of death, only the CATS and CONKO were able to stratify patients for VTE. (4) Conclusions: A new prediction model was generated to stratify lung cancer patients for VTE and mortality risk, where other published RAMs failed.

## 1. Introduction

Lung cancer is among the cancer types with the highest rates of venous thromboembolism (VTE), along with primary pancreatic, stomach, and brain tumors [1], with a reported incidence rate for all first VTE events equal to 39.2/1000 person-years as compared to 3/1000 person-years in the non-cancer population [2,3].

Different risk factors contribute to the increased risk of VTE in lung cancer, including patient (i.e., younger age, history of VTE, and comorbidities), cancer (i.e., advanced stage, adenocarcinoma subtype, proximity to the cancer diagnosis), and treatment-related factors (i.e., surgery, chemo- and hormone-therapy, erythropoiesis-stimulating agents, and anti-angiogenic therapy) [2,4,5]. In lung cancer patients, ALK/ROS1 translocations and KRAS mutations have recently been identified as potential VTE risk factors [6,7]. Overall, due to the interaction of all these risk factors, the VTE rate largely varies within the lung cancer population and in the same subject during the natural course of the disease. For example, patients with an advanced stage show the highest VTE rate compared to patients with localized disease [4], and chemotherapy, especially platinum-based, further increases this risk. No significant impact on this complication has been reported for radiotherapy [2], while data on immunotherapy are still inconclusive [8,9].

VTE causes important clinical consequences in cancer patients, including delays in antitumor treatments, hospitalization, and increased risk of VTE recurrence and bleeding during anticoagulant therapy [10]. More importantly, VTE adversely affects overall survival, representing the second cause of mortality after the cancer itself [11].

In the outpatient care setting, to avoid unnecessary anticoagulant exposure to subjects at low VTE risk, international scientific guidelines consider the use of pharmacological thromboprophylaxis only for patients at high risk of VTE, as estimated by risk assessment models (RAMs) [12], including the Khorana risk score (KRS) and the new-Vienna CATS risk score. Nevertheless, in patients with metastatic lung cancer initiating chemotherapy, the KRS is unable to identify subjects at high risk of VTE, while little information is available on the validity of the new-Vienna CATS score [13,14,15,16].

Therefore, the possibility to identify lung cancer patients at higher VTE risk during chemotherapy remains an important goal for precision medicine.

In addition to these two externally validated RAMs, others have been proposed over time, including the CONKO and the PROTECHT risk scores that attempted to improve the performance of KRS either by substituting the body mass index (BMI) parameter of the score with the performance status (i.e., the CONKO score), or by including the variable of high-risk chemotherapy types, i.e., cisplatin, carboplatin, or gemcitabine (i.e., the PROTECHT score) [17,18]. These two RAMs, however, still present some limitations when applied to the setting of metastatic lung cancer [19,20].

Of interest when considering specific cancer outcomes other than VTE, some of these RAMs outperform in the identification of subjects at a higher risk of disease progression or mortality [21,22,23]. We recently reported the inability of the KRS, as compared to the new-Vienna CATS score, to discriminate subjects at higher risk of VTE in the prospective HYPERCAN cohort of metastatic cancer patients, while both RAMs significantly stratified patients at higher risk of mortality [14].

Thereby, new RAMs for VTE, based on clinical and/or biological parameters that can help clinicians in deciding the anticoagulant strategy based on the individual risk of lung cancer, are needed. The tight and reciprocal interaction between cancer and hemostasis led us to investigate the role of hemostatic biomarkers in the prediction of VTE in lung cancer patients, especially in non-small cell lung cancer (NSCLC), which represents approximately 85% of all lung cancer cases, with adenocarcinoma and squamous cell carcinoma as the most common subtypes [24].

In this study, in a large prospective cohort of newly diagnosed metastatic NSCLC patients enrolled in the prospective, observational, multicenter HYPERCAN study (HYPERCAN study, Clinical Trials.gov ID# NCT02622815), we aimed to assess the utility of different hemostatic biomarkers in predicting for VTE and mortality within the first six months after diagnosis, during antitumor therapies. In addition, the KRS, new-Vienna CATS, PROTECHT, and CONKO RAMs were also assessed to predict the VTE risk and mortality in the same cohort of patients.

## 2. Materials and Methods

### 2.1. Study Population

The analysis included 568 newly diagnosed metastatic NSCLC adult patients prospectively enrolled in the HYPERCAN study (Clinical Trials.gov ID# NCT02622815) between May 2012 and July 2020 [25]. Patients were recruited at eight different Italian hospitals, and the study was coordinated by the Department of Immunohematology and Transfusion Medicine in the Hospital Papa Giovanni XXIII Bergamo, Italy. Inclusion criteria consisted of having newly diagnosed metastatic lung cancer (stage TXNXM1), age ≥ 18 years, a life expectancy >3 months, and being a candidate for first-line systemic chemotherapy. Exclusion criteria included having any acute medical illnesses, hospitalization, or therapeutic anticoagulation at enrollment.

Age, gender, BMI, Eastern Cooperative Oncology Group (ECOG) performance status, comorbidities, prophylactic use of anticoagulants (any reason other than cancer), use of antiplatelet drugs, tumor type and size, histological subtype, and tumor biological characteristics were recorded at the enrollment.

After inclusion, patients were followed up for at least five years, and clinical data on any thrombotic event, antitumor treatment, clinical response, and death within 6- months were recorded. Information on VTE collected during follow-up included the detection of symptomatic deep vein thrombosis (DVT); symptomatic non-fatal pulmonary embolism (PE); fatal PE; incidental proximal DVT (popliteal vein or higher); and incidental proximal PE (segmental arteries or larger). Incidental PE or DVT was defined as thrombosis reported during imaging testing performed for cancer staging and not for suspected VTE. The events were validated by the Independent Central Adjudication Committee to be included in the analysis. The median follow-up time of the cohort was 251 days (35–1873 days). Patients without an event were censored at six months from follow-up.

Local Ethics Committee (Comitato Etico della Provincia di Bergamo, del. 146, 1 February 2012) approved the study protocol, and all patients provided informed written consent for data recording, collection, and storage of blood samples, and allowed regular monitoring, statistical analysis, and publication of results. The study was conducted following the last revision of the Helsinki Declaration.

### 2.2. Blood Collection and Plasma Preparation

Peripheral venous blood was drawn into vacutainer tubes of 6 mL containing 0.109 M Na3 citrate (9:1 *v*/*v*; Becton Dickinson, Vacutainer, Plymouth, UK). Within 2 h of blood collection, platelet-poor plasma was obtained by two-step centrifugation at 3000× *g* for 10 min at room temperature and stored at <80 °C. The samples were all tested at enrollment for the hemostatic biomarker study at the Laboratory of Hemostasis and Thrombosis (Hospital Papa Giovanni XXIII, Bergamo, Italy) and assessed within a median time of 71 days from blood sample collection. The procedure of blood sample obtention, management, and storage was performed as described elsewhere [26].

### 2.3. Hemostatic Biomarkers

Plasma levels of fibrinogen (QFA thrombin, Werfen Group, Milan, Italy) and FVIII coagulant activity (HemosIL FVIII:c, Werfen Group) were measured according to manufacturer procedure, on an automated coagulometer analyzer (ACL TOP500, Werfen Group). Plasma levels of D-dimer (STA Liatest D-Di PLUS), protein C activity (PC, STA-STAChrom Protein C), and free protein S (free-PS, STA-Liatest Free Protein S) were measured on the STA Compact Max 3 coagulation analyzer. Plasma levels of prothrombin fragment 1 + 2 (F1 + 2) were measured using a commercially available ELISA (Enzygnost^®^, Siemens Healthcare Diagnostics, Munich, Germany).

### 2.4. Thrombin Generation (TG) Assay

TG was performed in all free-platelet plasma samples tested in duplicate by the calibrated automated thrombogram method at 5 pM Tissue Factor (TF) and 4 µM phospholipids (CAT assay, Stago, Maastricht, The Netherlands) [27]. The following parameters of the TG curve were considered: lag time (in minutes: time from test triggering to signal detection), time to peak (ttP; in minutes: time necessary for thrombin concentration to reach its maximal value), peak height (in nM: maximal thrombin concentration), and ETP (endogenous thrombin potential, in nM*min: area under the thrombin time concentration curve).

The reference intervals for the hemostatic biomarkers and thrombin generation were internally generated from a group of 200 apparently healthy controls (170 females; 30 males) free of cardiovascular disease, thrombotic or bleeding disorders, diabetes, cancer, or infectious diseases and were not taking antiplatelet or anti-inflammatory drugs in the last 10 days before blood sampling. The median age was 49 years (range 35–64 years).

### 2.5. Study Outcomes

The primary endpoint of the present analysis study was the occurrence of an objectively confirmed clinical VTE (i.e., confirmed by duplex sonography, phlebography, computerized tomography, or ventilation-perfusion lung scan) or death within 6 months of enrollment.

### 2.6. Statistical Analysis

According to their distribution, categorical variables were summarized as frequencies and proportions, and continuous variables as median with 5th–95th percentile range or mean with standard deviation (SD). Normally and non−normally distributed quantitative data were compared using the unpaired Student’s *t*−test and Mann–Whitney U test, respectively, while Chi2 was used to compare categorical data. Predictors of a 6-month VTE were identified by the competitive multivariate Fine–Gray proportional hazard regression model, considering death from any cause as the competing risk (CR) of interest (Fine-Gray Method [FGM], CR methodology, stcrreg STATA). A first approach by univariate analysis was carried out, considering all laboratory and clinical parameters as possible predictors to screen out variables with a *p*-value < 0.1. Further, a backward variable selection was applied, obtaining a proportional subdistribution-hazard ratio (SHR) of the significant (*p* < 0.05) variables for the multivariable analysis. The model obtained by the competitive multivariable analysis was then used to create a score by the percentiles of the continuous variable, together with the dichotomous variable. The discriminatory accuracy of the score was assessed by evaluating the area under the receiver operating curve (AUC, ROC). The bootstrap-based optimism correction method further assessed the model’s predictive ability [28,29]. Calibration plots for VTE were also applied to evaluate the performance of the model by the observed and predicted event (pmcalplot, STATA) (Figure S1, Supplemental Material), as recommended in the TRIPOD guidelines [30].

Competitive univariate Cox proportional hazard regression was applied to evaluate the predictive role of the different variables for 6-month VTE and death of the published scores (KRS, new-Vienna CATS, PROTECHT, and CONKO score). The Kaplan–Meier method was used to estimate survival functions, assuming the day of study enrollment as baseline time, and compared by log-rank test. The accuracy of the models (i.e., sensitivity, specificity, positive and negative predictive values) was evaluated. The statistical analysis was performed using StataCorp. 2019. Stata Statistical Software: Release 16 (StataCorp LLC, College Station, TX, USA).

### 2.7. KRS, New-Vienna CATS, PROTECHT, and CONKO Score Calculation

Based on the data available at enrollment, the KRS, new-Vienna CATS, PROTECHT, and CONKO scores were assessed. The KRS was calculated by clinical and complete blood cell count data collected at enrollment into the study before starting chemotherapy. This RAM assigns 1 point for NSCLC tumor type and 1 point to each of the following conditions: platelet count > 350 × 10^9^/L, leukocyte count > 11 × 10^9^/L, hemoglobin < 10 g/dL or use of erythropoietin stimulating agent, and a BMI ≥ 35 kg/m^2^ [31]. Patients were then classified as “low-risk” if the total score was <2 points or “intermediate-high risk” if the total score was ≥2 points, according to current guidelines [12,32]. Based on the tumor site and the continuous value of D-dimer, the new-Vienna CATS score was calculated according to the published formula to obtain the individual VTE risk, considering our cohort with a high-risk tumor site. The patients were then stratified according to a predefined risk set at 10%, which corresponds to 100 points by the nomogram [33]. We additionally used a second cut-off set of 5% (60 points), according to our last publication, which considered this value as a high enough risk, to give a guideline to start thromboprophylaxis [14].

The PROTECHT score was calculated with the variables of the KRS, adding 1 point for the patients treated with platinum or gemcitabine as a single agent or 2 points for those treated with platinum plus gemcitabine. Patients were classified as “low-intermediate-risk” if the score was <2 points or “high-risk” if the score was ≥3 points [17]. Finally, the CONKO score was calculated with the variables of the KRS, substituting BMI with the World Health Organization (WHO) performance status. A WHO performance status ≥2 adds 1 point to the score, and patients were classified as “intermediate risk” if the score is <2 points or “high risk” if the score is ≥3 points [18].

## 3. Results

### 3.1. General Characteristics of the Study Cohort

Table 1 shows the characteristics of the study population, which consisted of 568 NSCLC patients. The median age of the cohort was 65 years, the majority were male, and most had an ECOG performance between 0 and 1. The predominant NSCLC histological subtype was adenocarcinoma, followed by squamous cell carcinoma. Three hundred patients had two or more metastatic sites. The metastatic site was mainly intrathoracic (71%), followed by osseous (33%), encephalic (22%), and suprarenal (16%).

Median BMI was 24.5 kg/m^2^; current smoking was present in 34% of the patients, while 44% had a smoking history. Comorbidities were present in 76% of the patients. At enrollment, 19% of patients were taking antiplatelet agents in primary prophylaxis for cardiovascular risk or secondary prevention for previous arterial thrombosis (16% aspirin, 2% clopidogrel, and 1% ticlopidine); 6% were on prophylactic anticoagulation with low-molecular-weight heparin (LMWH).

After enrollment, patients started antitumor treatment that consisted of a platinum- or gemcitabine-based regimen in 66%, combined platinum with gemcitabine regimen in 22%, or another regimen in 9%. Furthermore, immunotherapy and target therapy were used in 15% and 10% of patients, respectively, and 49% in radiotherapy.

### 3.2. Thromboembolic Events and Mortality during Follow-Up

Within the first 6 months from enrollment, 62 patients developed a VTE with a crude cumulative incidence of 12% (95% CI 10–16%) in a median time of 64 days (95% CI 18–154). The median age of patients who experienced a VTE was significantly (*p* = 0.026) lower than those who remained VTE-free (62.6 ± 8.9 vs. 65.5 ± 9.7 years). The type of VTE was isolated PE in 30 subjects (3 massive and 1 fatal), isolated DVT in 23, and PE with DVT in 9. Among the 62 patients who developed VTE, three were on antiplatelet treatment, and five were on prophylactic LMWH. During the same period, 167 deaths occurred, with a cumulative incidence of 31% (95% CI 27–35), in a median time of 106 days (range 12–179 days). No patients were lost at follow-up during this 6-month analysis. Furthermore, VTE occurrence was associated with a significantly higher risk of death at 6 months (VTE vs. no VTE: 42 vs. 28%; log-rank *p* < 0.001).

### 3.3. Hemostatic Biomarkers and Thrombin Generation

As reported in Table 2, univariate analysis showed that patients who developed VTE were characterized by significantly (*p* < 0.05) higher pre-chemotherapy plasma levels of FVIII, PC, D-dimer, and F1 + 2 compared to patients who remained VTE-free. Furthermore, VTE patients displayed higher TG peak (*p* < 0.001) and shorter lag-time and ttP (*p* < 0.05) compared to VTE-free patients, while no differences were found in ETP values.

Non-survivor patients (Table 2) had significantly (*p* < 0.001) higher pre-chemotherapy levels of D-dimer, fibrinogen, and FVIII as well as significantly (*p* < 0.05) prolonged TG lag-time and higher TG peak compared to patients who survived during the first 6 months of follow-up after enrollment.

### 3.4. Clinical and Laboratory Predictors of VTE

Among the clinical characteristics, the Fine and Grey univariable analysis identified an ECOG = 2 (SHR 2.21, 95% CI 1.11–4.37; *p* = 0.023), lower age (SHR 0.97, 0.95–0.99; *p* = 0.015), and antiplatelet treatment (SHR 0.29, 0.06–0.63; *p* = 0.006) as independent predictive factors for VTE, also after 1000-bootstrapping resampling-based approach. By the same analysis among laboratory biomarkers, FVIII, PC, D-dimer, and TG peak were identified as independent predictive factors for VTE (Table S1A, Supplemental Material).

After multivariable regression backward elimination of the not significant variables, we found that D-dimer (SHR 1.124, 95% CI 1.066–1.183; *p* < 0.001) and ECOG = 2 (SHR 2.265, 95% CI 1.139–4.502; *p* = 0.020) were still independent risk factors for VTE. By a 1000-bootstrap-based model correction, we confirmed the result of the multivariate analysis.

**Figure 1:**
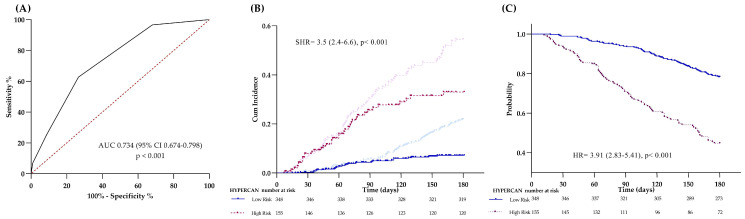
HYPERCAN score. (**A**) Receiver operating curve (ROC) of the predictive accuracy of the continuous model HYPERCAN for VTE diagnosis. (**B**) Crude cumulative incidence of VTE by Kaplan-Meier stratified in low- and high-risk by the HYPERCAN score; the light lines correspond to the estimation of the competing event for each group. (**C**) A 6 month-death by Kaplan Meier stratified in low- and high-risk according to the HYPERCAN score.

Therefore, we used these two parameters to generate the lung-HYPERCAN VTE risk score. First, four ranges of D-dimer concentrations were identified by the 25th, 75th, and 90th percentiles based on D-dimer distribution in the overall cohort of patients. Increasing points were then assigned to each D-dimer range from 0 to 3 points: i.e., <500 ng/mL (0 points), 500–1500 ng/mL (1 point), 1500–4000 ng/mL (2 points), and >4000 ng/mL (3 points). Thereafter, 1 point was assigned in case of an ECOG = 2 (Table 3). By this score (ROC AUC 0.734, *p* < 0.001) (Figure 1A), patients were stratified into two risk categories with a cumulative incidence of VTE of 6% (95% CI 4–10%) and 25% (24–42), in the low- and high-risk group (log-rank *p* < 0.001), respectively [SHR 3.5 (95% CI 2.4–6.6); *p* < 0.001] (Figure 1B).

### 3.5. Clinical and Laboratory Predictors of OS

By Cox regression univariable analysis, the presence of >1 metastatic site (HR 1.8, 95% CI 1.28–2.42; *p* < 0.001), radiotherapy (HR 1.76, 1.29–2.41; *p* < 0.001), and an ECOG = 2 (HR 3.84, 2.6–5.6; *p* < 0.001) were identified as predictive clinical factors for mortality at 6 months from enrollment. Leukocyte count, hemoglobin levels, FVIII, D-dimer, and TG peak were identified as independent risk factors among laboratory biomarkers (Table S1B, Supplemental Material).

By the multivariable analysis, an ECOG = 2 (HR 4.12, 95% CI 2.78–6.10; *p* < 0.001), and D-dimer (HR 1.001, 1.000–1.002; *p* < 0.001) remained as significant predictive factors for 6-month mortality risk. Therefore, by the same risk score provided for VTE patients, we stratified patients into 2 risk groups with cumulative incidences of death of 19% (95% CI 15–23%) in the low- versus 55% (47–63%) in the high-risk group category (log-rank *p* < 0.001), [HR 3.91(95% CI 2.83–5.41); *p* < 0.001] (Figure 1C).

### 3.6. Published RAMS for VTE and Mortality Risk Prediction

**Figure 2:**
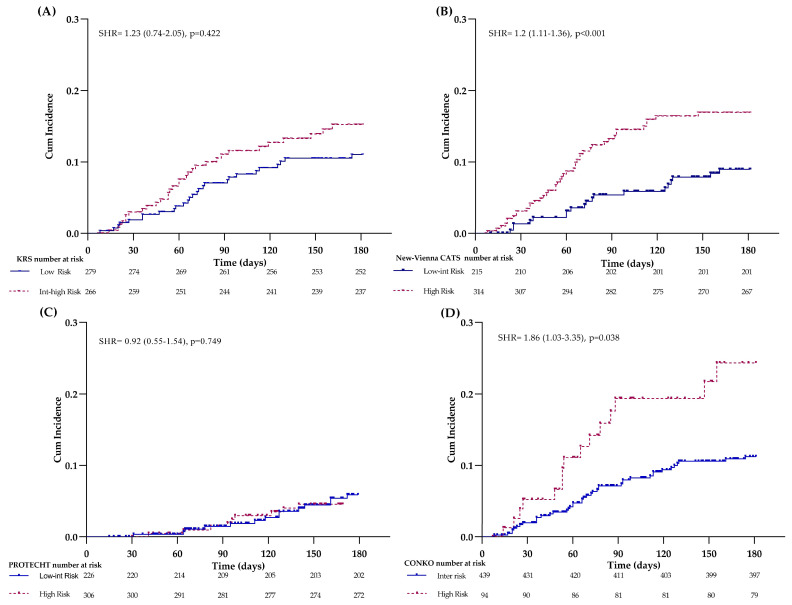
Venous thromboembolism (VTE) incidence by RAMs. Kaplan-Meier for the cumulative incidence of VTE by (**A**) KRS, (**B**) New-Vienna CAT score, (**C**) PROTECHT score, and (**D**) CONKO score.

**Figure 3:**
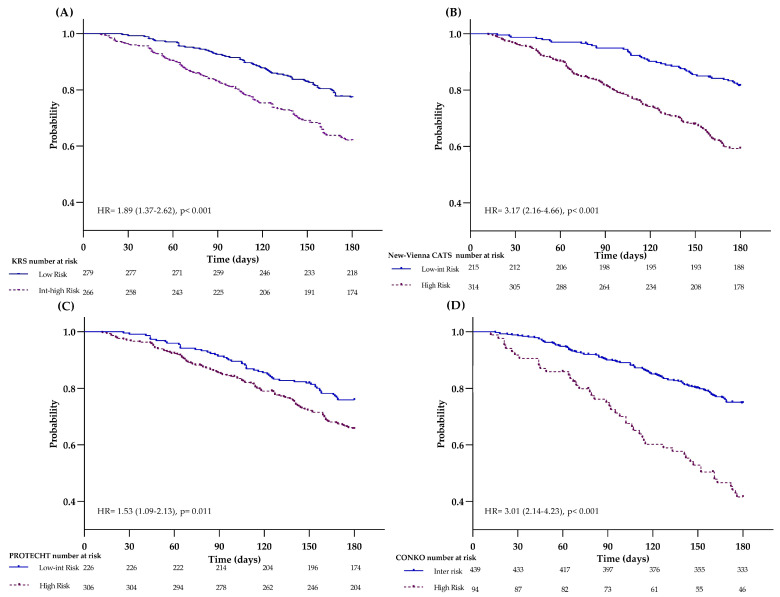
Overall survival (OS) by RAMs. Kaplan-Meier for 6-month OS by (**A**) KRS, (**B**) New-Vienna CAT score, (**C**) PROTECHT score, and (**D**) CONKO score.

The KRS was applied to 545 patients in the cohort. According to the parameters provided by this score, 2% of patients had a BMI ≥ 35 kg/m^2^, 33% had a leukocyte count >11 × 10^9^/L, 6% had a hemoglobin level <10 g/dL, and 25% had a platelet count >350 × 10^9^/L. A total of 461 patients scored ≤ 2 points and were therefore classified as low-risk, while 84 patients scored > 2 and were classified as intermediate-high risk. The crude VTE cumulative incidence was 11% (95% CI 9–15%) in the low- and 16% (9–30%) in the intermediate-high-risk group, with no significant difference between the two risk categories (log-rank *p* = 0.089), [SHR 1.23 (0.74–2.05); *p* = 0.422] (Figure 2A). Differently, the KRS significantly stratified the cohort at different risks of death, providing cumulative incidences of 26% (95% CI 22–30) vs. 49% (39–62%) (log-rank *p* < 0.001) in the low- vs. intermediate-high-risk group, respectively [HR 1.89 (1.37–2.62), *p*< 0.001] (Figure 3A).

The new Vienna-CATS score was applied to the cohort according to the published nomogram to calculate the individual risk of VTE. Then, patients were categorized as low-intermediate- or high-risk according to the 100-point cut-off scale, set at a VTE cumulative incidence of 10%. By this cut-off value, the 6-month cumulative incidence of VTE was not statistically different (*p* = 0.233) between the low-intermediate- vs. the high-risk group [12% (95% CI 10–16) vs. 20% (6–58)]. Differently, by the 60-point cut-off value (VTE incidence of 5%), our cohort displayed a cumulative incidence of VTE of 9% in the low-intermediate- vs. 14% in the high-risk group, *p* = 0.008, [SHR 1.2 (1.11–1.36), *p* < 0.001] (Figure 2B). Concerning mortality, by the 100-point cut-off, the 6-month cumulative incidence of death was 30% vs. 50% in the low-intermediate- vs. high-risk (*p* = 0.046) respectively [HR 2.41 (0.99–5.87); *p* = 0.054], while by the 60-point cut-off, the cumulative incidence of death was 15% vs. 40% in the low- intermediate- vs. the high-risk, respectively [HR 3.17 (2.16–4.66); *p* < 0.001] (Figure 3B).

The application of the PROTECHT score in 532 patients classified 226 patients at low-intermediate-risk and 306 patients at high risk. The VTE cumulative incidence was 11% in the low-intermediate- and 12% in the high-risk group, log-rank *p* = 0.730 [SHR 0.98 (0.55–1.54); *p* = 0.749] (Figure 2C). The same score applied for mortality significantly stratified (log-rank *p* = 0.012) patients at low-intermediate-risk (cumulative incidence of 24%) and high-risk (cumulative incidence of 34%) for death [HR1.53 (1.09–2.13); *p* = 0.011] (Figure 3C).

Finally, by applying the CONKO score, 439 patients were categorized in the intermediate- and 94 patients in the high-risk group. The cumulative incidence of VTE was 10% in the intermediate- and 19% in the high-risk group (log-rank *p* = 0.004) [SHR 1.86 (1.03–3.35); *p* = 0.038] (Figure 2D). In addition, by this score, a significant difference in the cumulative incidences of death was found between the intermediate vs. high-risk group (25% vs. 57%; log-rank *p* < 0.001) [HR 3.01 (2.14–4.23); *p*< 0.001] (Figure 3D).

To avoid bias due to the different number of patients included in each score, the RAMs were applied to the sub-group of patients with complete parameters for the 5 scores. The results showed no significant changes in the performance of all the scores (Table S2, Supplemental Material).

### 3.7. Accuracy of RAMs for VTE and Mortality

Table 4 shows the sensibility, specificity, positive predictive value (PPV), and negative predictive value (NPV) of the different RAMs evaluated for both VTE and mortality prediction.

Among the three RAMs that were able to significantly stratify for VTE risk in our patients’ cohort, both the new-Vienna CATS and HYPERCAN scores showed the highest sensitivity (70 and 63%, respectively), and NPV (93 and 92%, respectively) for VTE.

Regarding mortality risk prediction, PROTECHT, and new-Vienna CATS RAMs displayed better sensitivity (66 and 79%, respectively), while the HYPERCAN, KRS, and CONKO RAMs showed better specificity (80 and 90%, respectively).

## 4. Discussion

Lung cancer is a common malignancy with a clinically significant risk of VTE during chemotherapy but an unacceptable bleeding rate when pharmacological prophylaxis is applied indiscriminately to all patients [34]. Therefore, we try to develop a prediction model for VTE and death, starting from data from a large prospective observational cohort of 568 newly diagnosed metastatic NSCLC patients. Different available VTE RAMs for cancer patients were also tested. Data analyzed included clinical and routine laboratory parameters collected at enrollment and the levels of a series of hemostatic biomarkers that included FVIII, fibrinogen, D-dimer, free-PS, PC, F1 + 2, and TG parameters [25].

After 6 months from enrollment, 62 patients experienced a VTE, while 167 died. Due to the high mortality rate, the incidence of VTE was calculated considering all causes of death as a competing event. According to this analysis, a 6-month VTE cumulative incidence of 12% was found, in line with the rates reported by other prospective studies in lung cancer patients [35,36]. Notably, in our study, VTE was also associated with a significant two-fold risk of mortality, a finding initially reported by Sorensen et al. using a linked Danish database [37] and then by others in specific cancer types [38,39,40].

Our analysis of demographic and clinical characteristics showed that the group of patients who developed VTE was younger compared to those who remained VTE-free. Furthermore, by competitive risk analysis, younger age was identified as an independent risk factor for VTE (SHR 0.97; 95% CI 0.95–0.99), as previously reported by a population-based study of patients with NSCLC [4], and by a study of 673 hospitalized lung cancer patients, where younger age (<60 years) was associated with a higher risk of pulmonary embolism [41]. Commonly, older age is a main risk factor for VTE, and most patients with VTE are over 65; therefore, our observation, together with those from the aforementioned studies, goes in the opposite direction. A more aggressive cancer disease in younger patients might likely be responsible for this finding [42,43].

In addition to younger age, our data identified an ECOG performance of 2 as an independent risk factor for VTE. Poor performance status has been repeatedly associated with an increased risk of VTE in patients with cancer [18], especially when associated with immobility and hospitalization [20]. In the setting of ambulatory cancer patients, a post hoc analysis from the Hokusai-VTE study that evaluated 652 patients with cancer-associated PE, an ECOG of 2, was a predictor of VTE recurrence and mortality [44]. Therefore, our data further support the role of a poor ECOG as a risk factor for VTE in the outpatient setting.

Of interest, our study shows that, by univariable analysis, the use of aspirin was a protective factor for VTE occurrence, as previously observed in several surgical and non-surgical conditions [45]. In cancer, aspirin is utilized to prevent both arterial and venous thrombosis in multiple myeloma and myeloproliferative neoplasms [46,47]; fewer and conflicting data are available on its efficacy in other cancer types [48,49]. Our observation is of potential interest; however, it should be taken with the limitation that our study was not designed to test the efficacy of antiplatelet drugs.

The evaluation of the hemostatic biomarkers at enrollment showed in the overall cohort of patients a significant increase in the median plasma levels of FVIII, fibrinogen, D-dimer, and F1 + 2 as compared to normal reference values, underlying the well-known hypercoagulable state associated with cancer [50]. Remarkably, we also found significantly elevated levels of TG peak together with a shorter lag-time and time to peak, altogether indicating an increased TG potential. Finally, patients who experienced VTE showed significantly increased plasma levels of D-dimer, F1 + 2, FVIII, PC, and TG peaks, compared to patients who remained VTE-free during the same follow-up period.

To identify the most significant parameters associated with VTE development, a competitive multivariate regression analysis that included clinical and hemostatic biomarkers was performed with the identification of D-dimer and ECOG = 2 as the best independent risk factors for VTE. Based on these results, we generated the lung-HYPERCAN risk score for the prediction of VTE that included both D-dimer and ECOG. By this score, the cumulative incidence of VTE was 6% in the low- and 25% in the high-risk group (log-rank *p* < 0.001).

When analyzing data with mortality, among the clinical parameters, an ECOG = 2, the presence of more than one metastatic site, and the use of radiotherapy were identified as independent risk factors for death. These last features are suggestive of the presence of a major tumor burden and, consequently, a worse prognosis.

Among the hemostatic biomarkers, D-dimer, fibrinogen, FVIII, and TG peak were found significantly elevated in patients who died compared with patients who survived. However, after multivariable analysis, only D-dimer and ECOG remained significantly associated with mortality. Therefore, the same lung-HYPERCAN score was applied to overall survival providing a cumulative incidence of death of 19% and 55% in the low- and high-risk groups, respectively (log-rank *p* < 0.001).

In our prospective cohort, we also tested the performance of four published RAMs for VTE risk stratification., i.e., the KRS, the new-Vienna, the PROTECHT, and the CONKO scores. Among these, only the new-Vienna CATS (9 vs. 14%) and the CONKO (10 vs. 19%) scores significantly stratified patients for VTE risk, while the KRS and the PROTECHT failed. We have to consider that all four RAMs tested in the present study (i.e., KRS, CONKO, PROTECHT, and new-Vienna CATS) included the “tumor site” category in their scoring system, and all provided a fixed 1-point value (high-risk category) for the lung cancer site. Being the lung-HYPERCAN score specifically developed in lung cancer patients, the site category was not included in the scoring system.

The strength of the above RAMs, and mainly of the KRS, has been tested in different cancer cohorts. A meta-analysis regarding the KRS for the 6-month prediction of VTE in ambulatory cancer patients concluded that this score was of little help in the decision-making to start thromboprophylaxis [51]. More specifically, in lung cancer, RAMs have been evaluated in both retrospective and prospective studies [13,15,23,52,53]. In a retrospective cohort of 118 lung cancer patients, treatment with gemcitabine and a history of atrial fibrillation were the main risk factors for VTE, and the COMPASS-CAT score was the only RAM able to discriminate patients at high risk of VTE, compared to KRS, PROTECHT, and CONKO RAMs [52]. Another small retrospective study of 130 lung cancer patients confirmed the poor-discriminatory performance of the KRS for VTE prediction, while, interestingly, it performed well in predicting survival at 16 weeks [23]. Three prospective studies in lung cancer led to similar conclusions [13,15,53]. In a prospective cohort of 719 lung cancer patients with retrospectively adjudicated VTE, KRS was a predictor of mortality but not of VTE [13], as further confirmed by the analysis of the prospective observational cohort of 1980 lung cancer patients of the CANTARISK study [15]. Finally, the KRS did not perform well in lung cancer patients from the prospective cohort of the FRAGMATIC study [53]. Our results are in agreement with all these studies [13,15,23]. In addition, the results of all the evaluated scores, including the newly developed lung-HYPERCAN score, significantly stratify patients at different risks of death.

Our study presents some limitations. First, in accordance with the study design, VTE screening was not routinely performed by imaging techniques in asymptomatic patients. This procedure could lead to VTE underdiagnosis; however, on the other side, it limits the possibility of incurring surveillance bias. A second limitation is that our risk model has been only internally validated, and an external validation is warranted. Finally, due to the wide variety of D-dimer assays available from manufacturers and their laboratory applications, the performance of our risk model with D-dimer assays different from the one we used should be addressed in the future.

Despite not being externally validated, our score has a high potential for clinical application due to its simplicity (only two parameters) when compared to the other scores. The ECOG is a validated scale to define prognosis and guide treatment in cancer patients. D-dimer has been successfully incorporated into the new-Vienna CATS score, and our data further support its utility when combined with a clinical parameter. Indeed, as compared to the new-Vienna CATS score, our model shows greater specificity and PPV, with comparable sensitivity and NPV values, making it more performing in identifying lung cancer patients at high risk of VTE.

## 5. Conclusions

In conclusion, we generated and internally validated a RAM for the identification of metastatic lung cancer patients at higher risk of VTE, where other RAMS failed to provide significant results and death. Given its performance, our model could be used to select lung cancer patients at high risk for VTE and guide thromboprophylaxis use in clinical practice once it is externally validated.

## Figures and Tables

**Table 1:** General characteristics of the lung cancer patients’ cohort.

	Overall Cohort (n = 568)	VTE (n = 62)	Death (n = 167)
Male sex (n, %) Age (years, mean [SD]) BMI (kg/m^2^, mean [SD]) BMI ≥ 35kg/m^2^ (n, %) ECOG (n, %) 0 1 2	381 (67) 65 (9.5) 25 (4.3) 11 (2) 236 (42) 252 (44) 51 (9)	45 (73) 63 (8.9) 25 (4.4) 2 (13) 24 (39) 26 (42) 9 (15)	121 (73) 66 (9.7) 25 (4.3) 2 (1) 41 (25) 84 (50) 33 (20)
Smoking (n, %) Active Previous	194 (34) 250 (44)	22 (36) 28 (45)	53 (32) 79 (47)
Comorbidities ≥ 1 risk factor (n, %) Diabetes Hypertension Dyslipidemia Cardiopathy CVA history	432 (76) 61 (11) 241 (42) 86 (15) 48 (9) 10 (2)	45 (73) 8 (13) 26 (42) 5 (8) 4 (7) 1 (2)	129 (77) 22 (13) 83 (50) 24 (14) 12 (7) 2 (1)
Antithrombotic therapy (n, %) * Antiplatelet drugs Anticoagulants	108 (19) 35 (6)	3 (5) 5 (8)	30 (18) 14 (8)
Histological subtypes (n, %) Squamous Large-cell carcinoma Neuroendocrine Sarcomatoid Adenocarcinoma Mixed Mucinous Acinar Solid Papilar Non-differentiated Non classified	84 (15) 5 (1) 1 (0.2) 5 (1) 262 (46) 2 (0.4) 6 (1) 1 (0.2) 2 (0.4) 7 (1) 22 (4) 171 (30)	9 (15) 27 (44) 4 (6.5) 21 (34)	22 (13) 67 (40) 11 (7) 56 (34)
Metastatic site (n, %) Intrathoracic Bone Suprarenal Encephalic	401 (71) 189 (33) 92 (16) 124 (22)	41 (66) 22 (36) 11 (18) 20 (32)	121 (73) 65 (39) 41 (25) 44 (26)
Blood Count (median [95%CI]) Leukocyte, 10^9^/L Hemoglobin, g/dL Hematocrit, % Platelets, 10^9^/L	9.2 (4.6–19.2) 13.3 (9.8–15.5) 40 (31–47) 280 (145–507)	9.6 (3.0–26.9) 13.8 (9.8–15.4) 41 (31–46) 269 (125–431)	10.2 (4.8–27.7) 12.7 (9.3–15.1) 39 (29–46) 297 (127–524)
Chemotherapy (n, %) Platinum or Gemcitabine Platinum with Gemcitabine Other Immunotherapy (n, %) Target therapy (n, %) Radiotherapy (n, %)	377 (66) 127 (22) 48 (9) 85 (15) 54 (10) 281 (49)	41 (66) 15 (24) 6 (10) 10 (6) 10 (6) 40 (65)	112 (67) 40 (24) 15 (9) 9 (5) 7 (4) 70 (42)

Categorical data are presented as numbers (percentages) and shown for the entire study cohort at the enrollment. Blood cell count data are presented as median and 5th and 95th percentiles. NSCLC: non-small cell lung cancer, SD: standard deviation, BMI: body mass index, ECOG: Eastern Cooperative Oncology Group performance status, CVA: cerebrovascular accident, CI: confidential interval. * Prophylactic antithrombotic therapy at enrollment included low-molecular-weight heparin at prophylactic dose (LMWH) and antiplatelet that consisted of aspirin, clopidogrel, or ticlopidine.

**Table 2:** Hemostatic biomarkers and Thrombin generation according to VTE and death.

	Reference Value	VTE Free (n = 506)	VTE (n = 62)	*p*-Value	Survivors (n = 401)	Non-Survivors (n = 167)	*p*-Value
F1 + 2, pmol/L	215 (126–478)	255 (128–826)	338 (135–1122)	0.001	283 (144–824)	293 (141–1033)	0.151
D-dimer, ng/mL	110 (40–280)	1330 (170–5620)	2030 (160–8340)	0.002	620 (150–4800)	1230 (270–7600)	<0.001
Fibrinogen, mg/dL	150–400	475 (248–922)	444 (201–800)	0.231	475 (245–908)	524 (240–1061)	<0.001
FVIII, %	104 (73–145)	155 (75–295)	190 (92–362)	0.007	150 (71–250)	186 (101–280)	<0.001
Free PS, %	90 (70–120)	86 (59–116)	87 (58–116)	0.645	86 (59–116)	89 (59–110)	0.316
PC, %	98 (72–125)	120 (82–182)	132 (78–204)	0.025	120 (82–202)	124 (82–188)	0.951
TG lag time, min	3.1 (2.2–4.5)	3.3 (2.2–5.4)	3.1 (2.0–4.6)	0.023	3.2 (2.1–4.8)	3.3 (2.3–5.0)	0.042
TG ETP, nM*min	1702 (962–2601)	1847 (1228–2730)	1836 (1124–2993)	0.736	1853 (1279–2874)	1837 (1164–3033)	0.630
TG ttP, min	6.7 (4.7–8.8)	5.7 (4.1–8.6)	5.1 (3.7–7.4)	0.001	5.5 (4.0–8.2)	5.4 (4.1–8.1)	0.647
TG peak, nM	237 (128–404)	390 (210–598)	432 (278–592)	0.002	400 (206–591)	424 (244–642)	0.025

Data are presented as median and 5th and 95th percentiles. The *p*-value is the statistical significance of the Mann-Whitney U test to compare values between VTE patients versus VTE-free and non-survivor versus survivor patients. VTE: venous thromboembolism; F1 + 2: prothrombin fragment 1 + 2; FVIII: factor VIII; free PS: free protein S; PC: protein C; TG: thrombin generation; ETP: endogenous thrombin potential; ttP: time to peak.

**Table 3:** HYPERCAN score.

D-Dimer Levels (ng/mL)	Points
>4000	3
>1500–4000	2
500–1500	1
<500	0
**ECOG performance**	
2	1
0–1	0
Risk 0–1 point = low, ≥2 points high	

Data shows the final risk assessment model. By the points for D-dimer and ECOG = 2, the total score is divided into two risks: Low-risk 0–1 points, High-risk ≥ 2 points.

**Table 4:** Cumulative incidence of VTE and death, and accuracy of RAMs.

		6-Month VTE							6-Month Death						
RAM	Risk Category	Cumulative Incidence (95% CI)	Log-Rank (*p*-Value)	ROC AUC (*p*-Value)	Sen (%)	Spe (%)	PPV (%)	NPV (%)	Cumulative Incidence (95% CI)	Log-Rank (*p*-Value)	ROC AUC (*p*-Value)	Sen (%)	Spe (%)	PPV (%)	NPV (%)
HYPERCAN D-dimer/ECOG 2	Low High	6 (4–10) 25 (24–42)	< 0.001	0.734 (<0.001)	63	74	25	93	19 (15–23) 55 (47–63)	<0.001	0.726 (<0.001)	56	80	55	81
KRS Cancer site/BMI ≥ 35 kg/m^2^ Hemoglobin < 100g/L Platelet > 350 × 10^9^/L Leukocyte > 11 × 10^9^/L	Low Int-High	11 (9–15) 16 (9–30)	0.089	0.543 (0.290)	21	86	16	89	26 (22–30) 49 (39–62)	<0.001	0.609 (<0.001)	25	89	49	74
New-Vienna CATS * Cancer site/D-dimer	Low-Int High	9 (5–13) 14 (12–22)	0.008	0.642 (0.001)	70	43	14	92	15 (11–20) 40 (35–46)	<0.001	0.670 (<0.001)	79	50	40	85
PROTECHT Cancer site/BMI ≥ 35kg/m^2^ Hemoglobin < 100g/L Platelet > 350 × 10^9^/L Leukocyte > 11 × 10^9^/L Gemcitabine/Platinum	Low-Int High	11 (8–17) 12 (9–18)	0.730	0.527 (0.504)	59	42	12	89	24 (18–30) 34 (29–40)	0.012	0.584 (0.002)	66	46	34	76
CONKO Cancer site/WHO ≥ 2 Hemoglobin < 100g/L Platelet > 350 × 10^9^/L Leukocyte > 11 × 10^9^/L	Int High	10 (8–14) 19 (13–36)	0.004	0.558 (0.156)	26	85	19	90	25 (21–29) 57 (48–69)	<0.001	0.647 (<0.001)	31	90	56	75

Data shows the cumulative incidence of VTE and death of the five RAMs at different risk stratification. The accuracy of the RAMs by ROC curve and the sensibility, specificity, PPV, and NPV. RAM: risk assessment model; VTE: venous thromboembolism; HYPERCAN: hypercoagulation in cancer; KRS: Khorana risk score; BMI: body mass index; WHO: World Health Organization; ROC: receiver operating characteristics; AUC: area under the curve; Sen: sensitivity, Spe: specificity; PPV: positive predictive value; NPV: negative predictive value; Int: intermediate. * The New-Vienna CATS score set at a VTE cumulative incidence of 10%.

## Data Availability

The data presented in this study are available on request from the corresponding author.

## Supplementary Materials

The following supporting information can be downloaded at: https://www.mdpi.com/article/10.3390/cancers15184588/s1, Figure S1: Calibration Plots; Table S1: Univariable Cox regression analysis for (A) VTE and (B) death; Table S2: VTE risk estimation in all risk assessment models before and after elimination of the patients that could not be included at least in one of the 5 scores.
