# Supplementary material for: A New Risk Prediction Model for Venous Thromboembolism and Death in Ambulatory Lung Cancer Patients
Source: Cancers (Basel). 2023 Sep 15;15(18):4588. doi: 10.3390/cancers15184588 (PMC10527104; doi:10.3390/cancers15184588)
Supplement: Supplementary file 1 [file cancers-15-04588-s001.zip › Supplemental Table S1.docx]

*Supplemental material*

Table S1

1. Univariable analysis for VTE by Fine and Gray

|  | SHR | 95% CI | *p* value |
| --- | --- | --- | --- |
| Age (years)  ECOG= 2  Antiaggregant | 0.97  2.21  0.29 | 0.95-0.99  1.11-4.37  0.06-0.63 | **0.015**  **0.023**  **0.006** |
| F1+2, pmol  D-dimer, µg/mL  FVIII, %  PC, %  TG peak, nM | 1.08  1.07  1.05  1.09  1.22 | 1.02-1.13  1.02-1.12  1.02-1.09  1.02-1.16  1.06-1.37 | **0.003**  **0.004**  **0.002**  **0.012**  **0.005** |

1. Univariable analysis for death by Cox

|  | HR | 95% CI | *p* value |
| --- | --- | --- | --- |
| ECOG= 2  More than 1 metastatic site  Radiotherapy  Leucocyte, 10^9^/L  Hemoglobin, g/dL | 3.84  1.76  1.76  1.04  0.82 | 2.61-5.64  1.28-2.42  1.29-2.41  1.03-1.06  0.75-0.89 | **<0.001**  **<0.001**  **<0.001**  **< 0.001**  **< 0.001** |
| D-dimer, µg/mL  FVIII, %  TG peak, nM | 1.11  1.45  1.16 | 1.07-1.15  1.22-1.67  1.09-1.29 | **< 0.001**  **< 0.001**  **0.023** |
