# Supplementary material for: A New Risk Prediction Model for Venous Thromboembolism and Death in Ambulatory Lung Cancer Patients
Source: Cancers (Basel). 2023 Sep 15;15(18):4588. doi: 10.3390/cancers15184588 (PMC10527104; doi:10.3390/cancers15184588)
Supplement: Supplementary file 1 [file cancers-15-04588-s001.zip › Supplemental Table S1.pdf]

Supplemental material

Table S1

A) Univariable analysis for VTE by Fine and Gray

	SHR	95% CI	*p* value
Age (years)	0.97	0.95-0.99	**0.015**
ECOG= 2	2.21	1.11-4.37	**0.023**
Antiaggregant	0.29	0.06-0.63	**0.006**
F1+2, pmol	1.08	1.02-1.13	**0.003**
D-dimer, µg/mL	1.07	1.02-1.12	**0.004**
FVIII, %	1.05	1.02-1.09	**0.002**
PC, %	1.09	1.02-1.16	**0.012**
TG peak, nM	1.22	1.06-1.37	**0.005**

B) Univariable analysis for death by Cox

	HR	95% CI	*p* value
ECOG= 2	3.84	2.61-5.64	**<0.001**
More than 1 metastatic site	1.76	1.28-2.42	**<0.001**
Radiotherapy	1.76	1.29-2.41	**<0.001**
Leucocyte, 10^9/L	1.04	1.03-1.06	< **0.001**
Hemoglobin, g/dL	0.82	0.75-0.89	< **0.001**
D-dimer, µg/mL	1.11	1.07-1.15	< **0.001**
FVIII, %	1.45	1.22-1.67	< **0.001**
TG peak, nM	1.16	1.09-1.29	**0.023**
