# Supplementary material for: A New Risk Prediction Model for Venous Thromboembolism and Death in Ambulatory Lung Cancer Patients
Source: Cancers (Basel). 2023 Sep 15;15(18):4588. doi: 10.3390/cancers15184588 (PMC10527104; doi:10.3390/cancers15184588)
Supplement: Supplementary file 1 [file cancers-15-04588-s001.zip › Supplemental Table S2.pdf]

Supplemental material

Table S2. VTE risk estimation in all risk assessment models before and after elimination of the patients that could not be included at least in one of the 5 scores.

	VTE risk	VTE risk recalculation on the common group of patients
HYPERCAN	6 vs 25; p<0.001	6.6 vs 24; p<0.001
PROTECHT	11 vs 12; p=0.730	10.9 vs 12.26; p=0.536
CONKO	10 vs 19; p=0.004	10 vs 21; p=0.001
New-Vienna CATS	9 vs 14; p=0.008	7.96 vs 14.4; p=0.006
KRS	11 vs 16; p=0.089	10.1 vs 13.5; p=0.124
