# Supplementary figures and images for: A New Risk Prediction Model for Venous Thromboembolism and Death in Ambulatory Lung Cancer Patients
Source: Cancers (Basel). 2023 Sep 15;15(18):4588. doi: 10.3390/cancers15184588 (PMC10527104; doi:10.3390/cancers15184588)

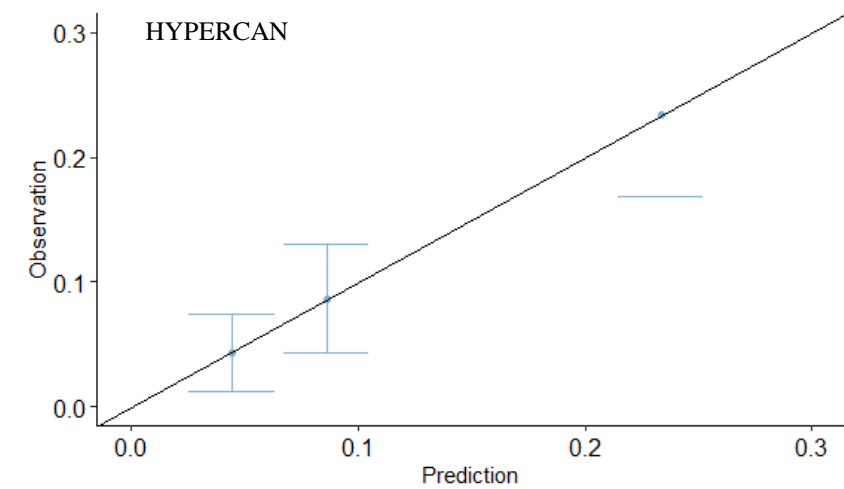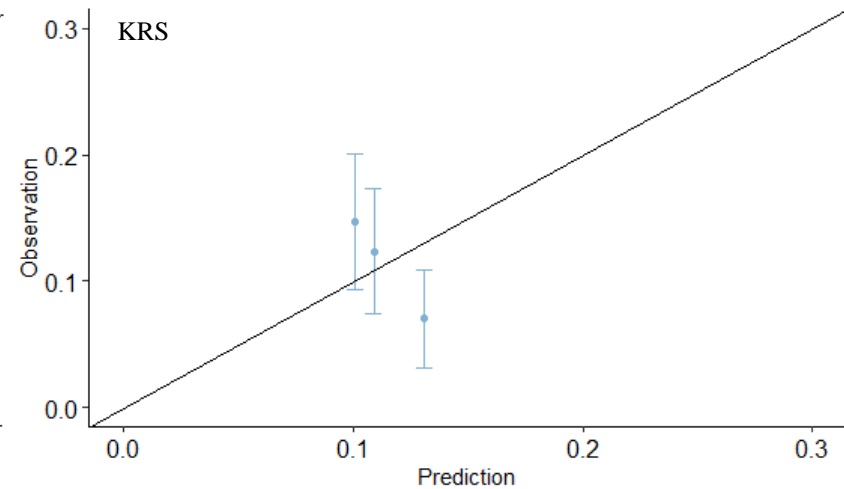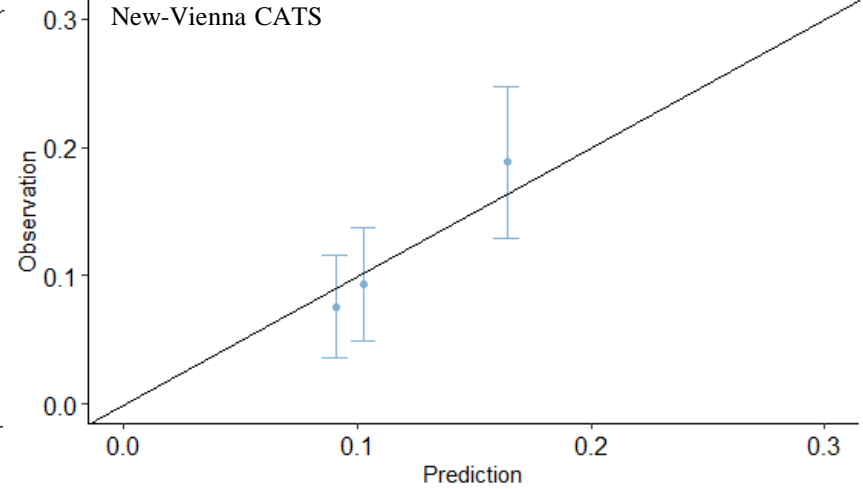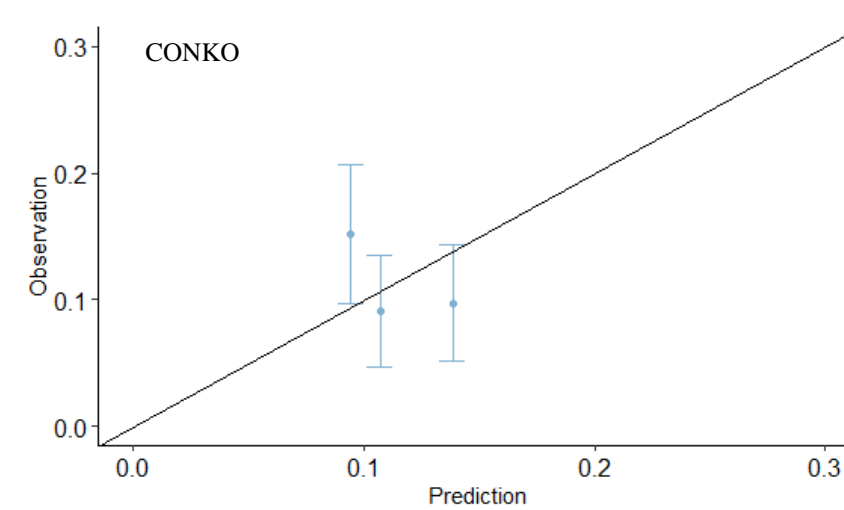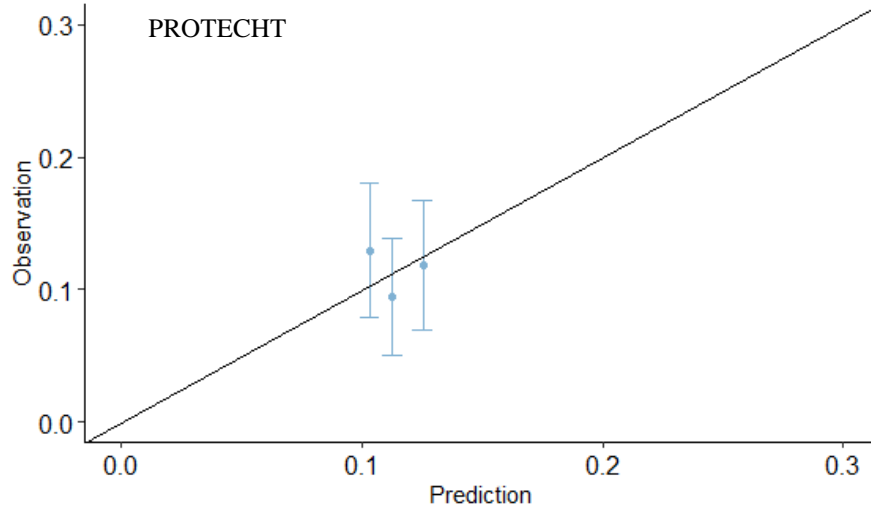

Supplement: Supplementary file 1 [file cancers-15-04588-s001.zip › Supplemental Figure S1.pdf]
